# Enhanced recovery of postoperative nursing for single-port thoracoscopic surgery in lung cancer patients

**DOI:** 10.3389/fonc.2023.1163338

**Published:** 2023-05-23

**Authors:** Xiufen Hu, Xiaodan He

**Affiliations:** ^1^ The No.1 Thoracic Surgery Ward, Liaoning Cancer Hospital & Institute, Shenyang, China; ^2^ The No. 1 Gynecological Ward, Liaoning Cancer Hospital & Institute, Shenyang, China

**Keywords:** single hole thoracoscope, lung cancer, enhanced recovery after surgery, nursing, treatment

## Abstract

Lung cancer is a common clinical malignant tumor, and the number of new lung cancer patients is increasing year by year. With the advancement of thoracoscopy technology and equipment, the scope of application of minimally invasive surgery has expanded to almost all types of lung cancer resection, making it the mainstream lung cancer resection surgery. Single-port thoracoscopic surgery provides evident advantages in terms of postoperative incision pain since only a single incision is required, and the surgical effect is similar to those of multi-hole thoracoscopic surgery and traditional thoracotomy. Although thoracoscopic surgery can effectively remove tumors, it nevertheless induces variable degrees of stress in lung cancer patients, which eventually limit lung function recovery. Rapid rehabilitation surgery can actively improve the prognosis of patients with different types of cancer and promote early recovery. This article reviews the research progress on rapid rehabilitation nursing in single-port thoracoscopic lung cancer surgery.

## 1 Introduction

Lung cancer is one of the malignant tumors with the highest incidence and mortality rates, making it the greatest threat to human health and life (1). Currently, the standard treatment for lung cancer is surgery along with postoperative chemotherapy or radiotherapy (2). However, most patients experience varying degrees of deterioration in quality of life after discharge from the hospital due to cardiopulmonary dysfunction, negative psychology, and adverse reactions from chemotherapy and radiotherapy, all of which harm their prognosis after surgery and may even shorten their survival period (3–5). In addition, chemotherapy may trigger hazardous effects in varying degrees (6, 7). Patients suffer from long-term pain and are under tremendous psychological stress (8, 9). They are physically and psychologically weary. Negative emotions such as anxiety and fear, in turn, affect their treatment efficacy and quality of life. To avoid being trapped in a treatment cycle, it is critical to strengthen nursing intervention during chemotherapy (10). Many studies have confirmed that lung cancer patients need additional assistance and care after being discharged from the hospital. Therefore, high-quality, efficient, continuing care should be given to patients to ensure and improve their quality of life after they return home.

Surgical resection is currently the most effective and important method for treating early stage lung cancer. Surgical methods are classified as thoracoscopic surgery and thoracoscopic surgery, while thoracoscopic surgery is further classified as multi-hole thoracoscopic surgery and single-port thoracoscopic surgery (11). Thoracoscopic surgery has been reported to minimize complications, improve postoperative quality of life, reduce postoperative discomfort, improve lung functions, shorten hospital stay, and hasten the return of patients to normal life (12–14). In the past 20 years, the popularity of minimally invasive techniques, namely video-assisted thoracoscopic surgery (VATS), has been continuously increasing and has been widely used in the treatment of cancer (15, 16). Since the first video-assisted thoracoscopic lobectomy was performed in the early 1990s. Previous studies have shown that for early non-small cell lung cancer, video-assisted thoracoscopy has significant benefits over traditional thoracotomy lobectomy, including shorter hospital stay, improved recovery, reduced perioperative complications, and improved long-term survival for selected patients (17–19). Thoracoscopic surgery usually includes 3–4 incisions. With the development of thoracoscopy technology and equipment, thoracoscopic surgery has progressed from being multiple-incision to double-incision surgery, and, finally, to single-incision thoracoscopic surgery, which is also known as single-port thoracoscopic surgery. **Table 1** depicts the various lung cancer treatment alternatives.

**Table 1:** The therapy approaches for lung cancer.

Therapy	Classification	Indications	Reference
Surgery	Lobectomy	The lesion is in a lobe	(20)
	Wedge or segment resection	Patients with wedge or segment resection with poor clinical cardiopulmonary function, small tumor lesions, and localized occurrence in a certain lung segment	(20)
	Sleeve lobectomy	Patients with upper lobe central lung cancer, especially those with compensatory cardiopulmonary function	(20)
	Extended resection	Invasive cancer	(20)
	Pneumonectomy	Central non-small cell lung cancer involving other tissue lesions	(20)
	Minimally invasive surgery	The lesion is in a lobe	(20)
	VATS	In patients with stage I and stage II lung cancer	(20)
Targeted therapy	Epidermal growth factor receptor tyrosine kinase inhibitor (EGFR-TKI)	EGFR mutant patients	(21)
	Anaplastic lymphoma kinase (ALK) inhibitor	ALK mutant patients	(22)
	Vascular endothelial growth factor (VEGF) inhibitor	all patients	(23)
Immunity therapy	Monoclonal antibody treatment	NSCLC	(24)
	Adoptive cellular immunotherapy	NSCLC	(24)
	Tumor vaccine	NSCLC	(25)
	Immune checkpoint targeted therapy	lung cancer	(26)
	Ipilimumab	SCLC	(27)
	Atezolizumab	SCLC	(28)
	Pembrolizumab	SCLC	(29)
	Nivolumab	SCLC	(30)
	Durvalumab	SCLC	(31)

In the 1990s, enhanced recovery after surgery (ERAS) was proposed (32). This concept optimizes the clinical pathways through evidence-based medicine, reduces traumatic stress, shortens hospital stays, and promotes early organ function recovery (33–35). The concept of ERAS has been progressively expanded in recent years, as have the perioperative nursing measures (36–38). Long-term hunger or dehydration, for example, should be avoided before surgery; carbohydrate beverages should be consumed before surgery, and early feeding and mobilization should be advocated. Unfortunately, there have not been many studies on intraoperative nursing measures that promote rapid postoperative recovery. As a result, this article focuses on the latest advancements in intraoperative nursing related to rapid postoperative recovery, which can promote patients’ early recovery after surgery and reduce the incidence of complications.

## 2 Application of single-port thoracoscopy in lung cancer surgery

### 2.1 The development of single-port thoracoscopic surgery

Hans Christian Jacobaeus can be considered as the first doctor to use a single port technique to enter the pleural cavity, perform biopsy and separate pleural adhesion. This event represents a widely accepted source of thoracoscopy or pleuroscopy examinations typically performed in autonomous breathing patients under local anesthesia (39). These surgeries are more diagnostic than therapeutic, with satisfactory diffusion, but they missed significant success. Until the 1970s, the role of thoracoscopy was still mainly limited to the diagnosis and treatment of pleural diseases (40). The modern backbone of single-port VAT has a relatively short but intense story, with a series of growing surgical achievements emphasizing its safety and effectiveness in both minor and major surgeries (41). Single-port VAT subtotal pneumonectomy follows the principles of open surgery in oncology, allowing for the dissection of hilar structures and complete radical lymph node resection for non-small cell lung cancer. Although the history of this technology is relatively short, its dissemination is rapid and stable. This is very rare and surprising, especially compared to other thoracotomy techniques or recent traditional thoracoscopy (42). Roviaro et al. reported the use of thoracoscopic lobectomy for the surgical treatment of lung cancer in 1992, and it was hailed as a revolutionary advancement and milestone in the surgical treatment of lung cancer (43, 44). The critical moment for single-port VATS was in June 2010, when Gonzalez Rivas underwent his first uniVATS lobectomy at La Coruna Hospital (43). They also reported the advancement of single-port thoracoscopic surgery in lung cancer radical resection. Since then, the second and most productive period of single port technology has begun, directly arising from multi port VATS. Initially, this technology was only used for lower lobectomy, but its rapid improvement is conducive to expanding the surgery to upper lobectomy, segmental resection, and total pneumonectomy (45, 46). The rapid development of single-port VAT technology has expanded indications and reduced contraindications. The success of all these surgeries depends on the skills of the surgeon and the availability of specific VATS instruments, which can be similarly applied to people of different body sizes. In the global prosperity, with the efforts and attempts of chest doctors, almost all common cancer surgeries can now be completed under a single hole thoracoscopy. **Table 2** lists the surgical categories of single hole thoracoscopy.

**Table 2:** The surgical categories of single-port thoracoscopy.

Surgical categories	Features	Reference
Single port thoracoscopic lobectomy	Anatomical lobectomy (subsequent sampling or dissection of mediastinal lymph nodes) is considered the current standard surgical treatment	(47)
Single port thoracoscopic segmentectomy	It is safe and feasible, can preserve the patient’s lung function to the greatest extent, and is suitable for stage Ia and Ib lung cancer, Patients with lung metastases and benign lung lesions. Especially suitable for elderly patients with poor lung function	(48)
Single-port thoracoscopic wedge resection of lung	A safe and effective method for diagnosing and treating isolated peripheral nodules	(47)
Single-port thoracoscopic sleeve resection	It is suitable for patients with central lung cancer invading the bronchus or blood vessels, and the lung tissue is preserved as much as possible	(48)
Single-port thoracoscopic pneumonectomy	It has potential advantages such as protecting the stability of the chest wall, reducing pain, reducing blood loss, shortening the hospital stay and returning to normal life activities as soon as possible	
Single port thoracoscopic surgery with subxiphoid approach	A safe, feasible and more minimally invasive surgery that can reduce postoperative pain and improve the quality of life of patients after surgery	

### 2.2 Application of single-port thoracoscopy

Gonzalez-Rivas et al. reported the world’s first single-port thoracoscopic lobectomy and mediastinal lymph node dissection in 2011 (49–51). Their report summarizes 222 cases of single-port radical resection of lung cancer performed at a single center, including relatively advanced lung cancer or complicated cases requiring preoperative adjuvant chemotherapy. The results revealed that only 3.6% of all patients were converted to two-port thoracoscopy or thoracotomy, with the average postoperative hospital stay being 3 days, and all patients recovered during the perioperative period. Single-port thoracoscopy is a safe and feasible treatment for lung cancer resection. The most prominent feature of a single-port laparoscope is that it only requires a 3–5-cm incision, does not stretch the ribs, and does not employ Trocar (52). All surgical instruments were accessed from this, and the surgery was completely performed under a thoracoscopic TV monitor. Patients undergoing single-port thoracoscopic surgery adopt the same lateral position as in traditional multi-port thoracoscopy. Many thoracic surgery clinicians have begun to adopt single-port thoracoscopy devices (12, 53).

### 2.3 Advantages and disadvantages of single-port thoracoscopic surgery

The single-operation hole incision design mainly eliminates the posterior axillary line incision while extending the anterior axillary line incision (49). All operating instruments including the scope are in and out of one operating hole. Meanwhile, after the anterior axillary incision is relatively extended, the chest wall muscles are less layered, allow easy stopping of bleeding, and possess high elasticity so that it does not cause further damage to the body, cause only slight postoperative pain, and has a negligible effect on patient’s sensation and movement (54, 55). In addition, when deciding where to perform the incision, we try to choose relatively concealed areas, such as the armpit or lower edge of the breast. However, the single-port thoracoscopy has its shortcomings: an operational hole, all the instruments are in and out of it, there is mutual interference between the instruments, and it is common for one instrument to enter and the other instruments to be unable to enter or move (18, 56). In addition, the fumes produced by the electrosurgical knife or electrocoagulation cannot be easily released. For the lesions toward the back or near the diaphragm, the exposure is poor, which complicates the surgery, and the instruments have to be repeatedly exchanged in and out, thereby increasing the operation time (57). Moreover, for patients with severe adhesions and intraoperative bleeding, operation may become unmanageable and difficult for beginners to grasp, and cause damage to the surrounding organs and tissues (57, 58).

### 2.4 Problems and prospects in the application of single-port thoracoscopic surgery

The goal of minimally invasive surgery is to alleviate discomfort and improve patients’ quality of life. The next approach in minimally invasive thoracic surgery is single-port thoracoscopic surgery. At present, more and more thoracic surgeons are experimenting with single-port thoracoscopic radical resection of lung cancer, transitioning from open to porous, traditional porous to a single port. This is the result of thoracic surgeons’ tireless pursuit of minimally invasive surgery. However, some problems remain, such as the short clinical application time and the lack of prospective randomized multi-center clinical research that confer poor reliability to the results. The existing thoracoscopy-related equipment is insufficient for single-port thoracoscopy operations and the surgical technique is difficult and risky. The learning curve is steep and difficult to master. With the accumulation of experience and the development of efficient equipment, improvement of the learning curve, and the support of more relevant, excellent papers, single-port thoracoscopy can be continuously improved and sublimated to hopefully demonstrate the incomparable advantages of traditional multi-holes and become the future of thoracic surgery for lung cancer resection. The standard surgical procedure has ushered in a new age of minimally invasive lung cancer surgery, benefiting more patients.

## 3 Concept and development of eras

### 3.1 The concept of ERAS

ERAS refers to the use of multidisciplinary treatment methods based on evidence-based medicine to optimize various medical behaviors and nursing measures during the perioperative period to decrease surgical stress, reduce complications, and accelerate patient recovery (34, 59, 60). The core principle here is to implement the best perioperative care measures based on evidence to achieve the goal of rapid recovery of patients. The ERAS concept involves the following 4 parts: multidisciplinary cooperation, a multi-modal approach to problem-solving, adopting a scientific, evidence-based approach to nursing programs, and applying interactive and continuous auditing in management (61–64).

### 3.2 Development of ERAS

Fast-track surgery (FTS) was initially proposed in the 1990s. Its goal is to facilitate quick and safe discharge of patients (65, 66). The main goal is to shorten the length of hospital stay. With the continuous updating of medical knowledge, FTS can gradually be replaced by ERAS, which focuses on the quality of patients’ rapid recovery, such as the reduction of postoperative stress response and the reduction of postoperative complications, which can accelerate patients’ physiology and psychological and social recovery (65–67). In 1997, Henrik Kehlet first described the concept of ERAS in colorectal surgery (68, 69). Over the years, it has developed into a multidisciplinary team approach, including surgeons, anesthesiologists, intensive care physicians, physical therapists, dietitian and nurses to participate in perioperative nursing of patients, and incorporate evidence-based protocols into clinical practice. This multimodal approach has been proven to shorten hospital stay, reduce surgical stress response, reduce incidence rate, and speed up rehabilitation. Subsequently, the ERAS society was established in 2010 and guidelines have been published for colorectal, bariatric surgery, gastrectomy, liver surgery and gynaecologic oncology. The implementation of ERAS protocols has decreased the cost of overall treatment without compromising outcomes (70). Research has demonstrated that compared to traditional care, postoperatively, patients have reduced intestinal obstruction and cardiopulmonary complications with shortened length of hospital stay (71). The first ERAS guidelines on colorectal cancer surgery were published in 2012, and this is the fourth update (72). ERAS guidelines on gynecology, gastrointestinal surgery, urology, and thoracic surgery were also issued during this period to regulate medical and nursing measures during the operation period with the aim of speeding up patient recovery and promoting the development of disciplines.

## 4 Eras application in the nursing of patients undergoing single-port thoracoscopic surgery for lung cancer

In the past decade, many studies have shown that ERAS in thoracic surgery has reduced the incidence of cardiopulmonary complications, reduced the use of opioids, minimized fluid overload, shortened hospital stay, and reduced hospital costs (73, 74). it has been reported that in 234 patients who underwent open lobectomy for cancer and concluded that ERAS reduced the incidence of complications from 50% to 37%, while there was no difference in readmission or emergency room visit rates (75). In another study targeting 2886 patients undergoing open and minimally invasive (VATS) pneumonectomy, Van Haren et al. concluded that ERAS can shorten hospital stay by one day, reduce pulmonary complications from 29% to 20%, and reduce cardiac complications from 18% to 12% (76). All these results showed that believe that many of the ERAS elements were already part of their standard care following VATS, and thus their new protocol may not have been significantly different enough to impact outcomes, supporting the application of ERAS in thoracic surgery (77).

Since its launch, single port VATS has become a viable alternative to the multi port VATS method for treating non-small cell lung cancer patients. Therefore, chest surgeons have been able to perform increasingly complex chest surgeries and incorporate this method into their surgical equipment (78). Combining minimally invasive technology with mature ERAS pathways can optimize hospitalization and treatment, especially for cancer patients (79). Therefore, establishing ERAS for single port VATS is worthwhile. Due to the fact that the incision of single port VATS is limited to one intercostal space and the length of the incision is smaller than that of multi-port VAT, it is necessary to reduce trauma to blood vessels, muscles, and nerves, as well as postoperative discomfort and abnormal chest sensation. The combination of single port VATS and ERAS may provide real basic benefits for patients in terms of rehabilitation, reducing postoperative incidence rate and improving quality of life (79). It is undeniable that an increasing number of papers have defined the economic benefits and rehabilitation efficiency of ERAS protocol in other surgical procedures and single port VATS patients. However, only a few reports have evaluated the comprehensive results. The application of ERAS could contribute to improving the quality of care, patient safety, and team efficiency and ultimately could save money.

### 4.1 Preoperative care

#### 4.1.1 Mental care and health

People’s needs for nursing care are becoming increasingly complex as medical models evolve. Exploring new medical care models has therefore become one of the hot research topics in the medical and health fields. Relevant literature research data suggest that several types of nursing models focus on psychological nursing and health education. When lung cancer patients experience the pressures of the disease and the high expense of therapy, their psychological pressure is relatively serious, mood swings are common, and they lack confidence in the treatment. When medical professionals perform medical and nursing services with a low level of patient cooperation, the treatment and nursing effects are compromised. Psychological care and health education for lung cancer patients are particularly important at this time. The research results suggest that intensive psychological care and health education for lung cancer patients can significantly improve the quality of life score (80). Foreign scholars believe that psychological care and health education for lung cancer patients might hasten postoperative recovery and reduce intraoperative stress (81). Some scholars believe that quitting smoking around 8 weeks before surgery can reduce the incidence of complications by about 35% (71). Strengthening communication with patients and their families, conducting targeted psychological interventions and pre-operative health education, and patiently explaining the disease mechanism and specific treatment methods to patients can effectively reduce the psychological pressure of lung cancer patients and improve treatment compliance.

#### 4.1.2 Intestinal preparation

Mechanical bowel preparation is a conventional form of bowel preparation (enema with strong oral laxatives). Routine bowel preparation has been demonstrated to aggravate patients’ preoperative dehydration and intraoperative stress response, resulting in a significant increase in the incidence of postoperative intestinal paralysis, which is not conducive to patients’ early recovery (82–84). Therefore, bowel preparation is not recommended. Some scholars compared the experimental group (without bowel preparation) and the control groups (with bowel preparation) to show that the incidence of complications (such as incision infection and lung infection) in the experimental group was not statistically different from that in the control group (72).

#### 4.1.3 Diet care

Traditional surgical operations may cause gastrointestinal discomfort in lung cancer patients, which may eventually affect the operation outcomes, aggravate the clinical pain of lung cancer patients, and cause complications in severe cases (85). Therefore, it is necessary to strictly control the diet of lung cancer patients before surgery. Due to the low physical fitness level of lung cancer patients, patients are recommended to consume high-protein liquid food a day before surgery, fast for 6 h before surgery, and drink 300–500 mL of glucose solution in the morning on the day of surgery. The foregoing procedures eliminate aspiration in lung cancer patients during the operation, while also preventing perioperative thirst and hypoglycemia, ensuring smooth operation, and preventing patients from feeling uncomfortable due to fasting and liquid intake.

### 4.2 Intraoperative care

#### 4.2.1 Intraoperative heat preservation

A normal temperature can effectively ensure a body’s normal metabolism. During the operation, the patient’s body temperature drops due to anesthesia, low ambient temperature in the operating room, or infusion factors. Intraoperative hypothermia increases the risk of postoperative wound infection rate, affecting patients’ blood coagulation function and prolonging their recovery time. To diminish the prognostic impact, lung cancer patients exhibit the following reactions during the postoperative recovery period: chills, as the first symptom, followed by arrhythmia and restlessness. There are several specific measures to keep the patient warm: first, reasonable use of thermal insulation pads (placing patients on a constant temperature thermal insulation pad to continuously increase the body surface temperature and reduce the body surface heat loss); second, active adjustment of the room temperature (set the temperature in the operating room) at 22–24°C; third, performing warm intravenous infusion or blood transfusion; fourth, reducing the body exposure of lung cancer patients during surgery; fifth, reducing the heat loss from the surgical infusion and using constant temperature saline to flush patients’ wound.

#### 4.2.2 Improved anesthesia

Improving the procedure of intraoperative anesthesia is an indispensable part of rapid rehabilitation surgery. The procedure involves adjusting short-acting analgesics (such as propofol), managing anesthetic injection timing and speed, and focusing on pain assessment. Adjusting the dose of anesthetic drugs depending on the results of the pain evaluation can significantly reduce clinical pain of lung cancer patients.

### 4.3 Postoperative care

#### 4.3.1 Get out of bed early

Getting out of bed early after surgery is an effective means to prevent postoperative complications. Long-term bed rest following surgery causes the following complications in lung cancer patients: muscle atrophy; muscle degeneration; deep vein thrombosis; and pulmonary complications. Early activities after surgery for lung cancer patients can be divided into the following stages: 1) 6–12 h after the operation, when the lung cancer patients are conscious and their physiological indicators are stable, the nursing staff can help them in performing deep breathing exercises and active and passive joint activities; 2) Taking a sitting position, 24–72 h after operation, increases the active movement of upper limbs and trunk; 3) Trying walking activities 72 h before discharge from hospital by gradually increasing the walking distance and speed and restoring the range of motion of the shoulder joint to that before surgery.

#### 4.3.2 Postoperative analgesia

Lung cancer patients often experience discomfort after surgery; therefore, hard breathing and coughing are severely restricted, which affects sputum production and increases the risk of lung infection. Moreover, pain can cause gastrointestinal discomfort in lung cancer patients, making it impossible for them to rest comfortably, leading to a series of negative psychological emotions; pain also restricts patients’ early activities after surgery. Therefore, the existence of pain has a significant impact on the postoperative recovery of lung cancer patients. Strengthening postoperative analgesia at this time can effectively relieve patients’ negative psychological emotions, prevent the occurrence of related complications, and enable patients to rest as soon as possible. Some scholars have proposed that self-controlled analgesia pumps can effectively relieve the pain of lung cancer patients undergoing thoracoscopic surgery.

#### 4.3.3 Postoperative catheter care

Insertion of various types of catheters after surgery increases the risk of infection and affects postoperative activities in patients with lung cancer. To avoid difficulties, it is vital to choose a reasonable drainage tube. Nursing staff needs to strictly control the extubation time when managing the chest drainage tube. A study revealed that removing the drainage tube following lobectomy with thoracoscopy is safer when the daily drainage volume is ≤500 mL. Some scholars also suggest removing the drainage tube when the daily drainage volume is ≤300 mL.

## 5 Issues and prospects

Problems while using ERAS to care for patients undergoing lobectomy are as follows: first, the ERAS concept of balanced analgesia emphasizes the use of non-opioids or limited opioid usage. However, these principles are often not clinically adopted, and medical staff should pay more attention to analgesia and key regional anesthetic techniques, as well as continue to research scientific and effective postoperative pain relief approaches. Second, several studies lack descriptions of crucial information such as patients’ general condition, type of surgery, postoperative activities, postoperative nutrition, and postoperative analgesia, making it impossible to analyze and judge the study results fully and objectively. Furthermore, since factors such as patients’ traditional concept, medical expense payment system, and hospital benefits impact the patients’ postoperative hospital stay, the role of confounding factors should be considered while using this indicator to quantify the effect of lobectomy. Moreover, taking postoperative complications as an essential indicator for ERAS, the impact of disease complications and surgical complications on the research results should be evaluated.

It has been found that the traditional general anesthesia VATS achieves lung surgery through tracheal intubation with unilateral lung ventilation. As an invasive device, tracheal intubation has caused many adverse reactions to the postoperative rehabilitation of patients. Non-intubated thoracoscopy has become the focus of attention for many scholars (86). Non-intubated VATS, also known as thoracoscopic surgery without tracheal intubation, is a new method of minimally invasive surgery that involves general anesthesia and natural ventilation. Previous study showed that the application of non intubated VATS can be better combined with ERAS strategy, leading to quickly recovery of postoperative patients (87). These results highlighting the potential clinical benefits from integrating non-intubated thoracoscopy with ERAS.

## 6 Conclusion

ERAS has gradually become a surgical development trend. ERAS nursing effectively combines psychological nursing, pain nursing, nutritional support, postoperative guidance, early eating and activities, and other nursing measures to provide surgical treatment with the support of evidence-based medicine. Medical care services should be aimed at providing better and more efficient care. Nursing methods compatible with ERAS are physically and psychologically closer to patients, embodying the concept of people-oriented medical care, and can effectively reduce postoperative complications, shorten the length of hospital stay, and reduce medical expenses. However, the ERAS concept’s applicability to patients undergoing lobectomy necessitates multidisciplinary collaboration. Clinical randomized controlled trials that are rigorous, standardized, and of high quality are still lacking. It is necessary to ensure medical staff training and encourage surgeons, anesthesiologists, nurses, and physical therapists to develop and test technologies or concepts that can promote recovery of patients undergoing lobectomy through multiple channels, as well as to pass more high-quality clinical trials in order to further verify the application effect of the ERAS concept in lobectomy. Similarly, health education for patients and their families should be improved to gain their cooperation in ERAS implementation. In addition, the safety issues of the ERAS concept in lobectomy cannot be ignored. It is not advisable to blindly practice ERAS. Researchers should carefully treat and implement it in the light of specific conditions and needs of patients.

## Author contributions

XiuH: Conceptualization, Methodology, Software Data curation, Writing- Original draft preparation. XiaH: Visualization, Investigation, Supervision. Writing- Reviewing and Editing. All authors contributed to the article and approved the submitted version.
